# Investigating the Current and Future Co-Occurrence of *Ambrosia artemisiifolia* and *Ophraella communa* in Europe through Ecological Modelling and Remote Sensing Data Analysis

**DOI:** 10.3390/ijerph16183416

**Published:** 2019-09-14

**Authors:** Mattia Iannella, Walter De Simone, Paola D’Alessandro, Giulia Console, Maurizio Biondi

**Affiliations:** Department of Life, Health & Environmental Sciences, University of L’Aquila, Via Vetoio Coppito, 67100 L’Aquila, Italy; mattia.iannella@univaq.it (M.I.); paola.dalessandro@univaq.it (P.D.); giulia.console@graduate.univaq.it (G.C.); maurizio.biondi@univaq.it (M.B.)

**Keywords:** *Ambrosia artemisiifolia*, *Ophraella communa*, ecological niche modelling, remote sensing, GIS analysis, biological control, Europe

## Abstract

The common ragweed *Ambrosia artemisiifolia* has spread throughout Europe since the 1800s, infesting croplands and causing severe allergic reactions. Recently, the ragweed leaf beetle *Ophraella communa* was found in Italy and Switzerland; considering that it feeds primarily on *A. artemisiifolia* in its invaded ranges, some projects started biological control of this invasive plant through the adventive beetle. In this context of a ‘double’ invasion, we assessed the influence of climate change on the spread of these alien species through ecological niche modelling. Considering that *A. artemisiifolia* mainly lives in agricultural and urbanized areas, we refined the models using satellite remote-sensing data; we also assessed the co-occurrence of the two species in these patches. *A. artemisiifolia* is predicted to expand more than *O. communa* in the future, with the medium and high classes of suitability of the former increasing more than the latter, resulting in lower efficacy for *O. communa* to potentially control *A. artemisiifolia* in agricultural and urbanized patches. Although a future assessment was performed through the 2018 land-cover data, the predictions we propose are intended to be a starting point for future assessments, considering that the possibility of a shrinkage of target patches is unlikely to occur.

## 1. Introduction

Invasive alien species (IAS) are a topic of major importance in many research areas, because of the countless implications they have on environment and human activities. IAS can alter ecosystem functioning by replacing, competing, or directly preying upon native species [1,2,3,4]. IAS can have a detrimental impact on agriculture, livestock farming, and, sometimes, human health [5,6,7], with significant impacts in terms of both social and economic costs for managing strategies [8,9,10,11,12]. 

The successful establishment of IAS depends on many factors, including abiotic and biotic interactions, as well as movement capabilities, concepts expressed in the biotic abiotic movement (BAM) diagram of Soberón and Peterson [13]. Recently, many papers investigated the response of IAS to climate change, with several studies indicating an increase of potential invasiveness, which goes along with the global change [14,15,16]. In this context, ecological niche models (ENMs) are a tool that can be used to infer the distribution of species in different spaces or times (e.g., [17,18,19,20,21]), even when dealing with IAS. In this case, models can be calibrated on native areas of the IAS and subsequently projected to the environmental conditions of the invaded ranges; this can also be achieved for future climatic conditions [1,22].

One of the most studied IAS is the common ragweed *Ambrosia artemisiifolia* L., an invasive plant from North America, which is known to be in Europe since the 19th century. It rapidly expanded and has become a weed of great importance in cultivated lands, mainly soybean, sunflower, maize, and sugar beet [23,24,25]. Common ragweed is also known for its high allergenicity, with several socio-economic consequences [26,27], and considerable effort has been dedicated to its control or even eradication [28,29,30,31,32,33].

Since 1995 [34], the ragweed leaf beetle *Ophraella communa* LeSage, another IAS from North America, has occurred in a “parallel” spread with respect to *Ambrosia artemisiifolia* in some Asian, first, and European countries, after. This leaf beetle species mainly feeds on *A. artemisiifolia*, causing heavy defoliation and decreasing the amount of pollen produced by the host plant [25]. Indeed, the co-occurrence of both species is important to biocontrol the invasion of *A. artemisiifolia* as stated by Buttenschøn et al. [35], Sun et al. [36], and Lommen et al. [37], especially for past failed attempts of biocontrol by *Zygogramma suturalis*. Further, *O. communa* seems to be a good candidate for the control of *A. artemisiifolia* in Asia and North America [38,39]. The control is also encouraged, considering that non-target plants occurring along with *A. artemisiifolia*, such as sunflower and other crops, are damaged in a smaller proportion with respect to the target weed [40,41]. 

Recently, the machine-learning algorithm implemented in Maxent [20] was used to infer the possible response of *A. artemisiifolia* to climate change scenarios [42,43,44,45], or for investigating co-occurrences between *A. artemisiifolia* and *O. communa* [36,46]. However, notwithstanding the high interest of these two IAS in the human health and agricultural research areas [37,47,48,49], few papers directly deal with the potential current and future distribution of these two species in the secondary ranges, especially in light of climate change [36,37,50]. These articles focus mainly on the co-occurrence of ragweed and its possible biological control agents through a biogeographic modeling approach (species distribution models), in East Asia [36] and Europe [37,50,51]. Although important information is provided, no specific inferences are made about the areas where *A. artemisiifolia* is usually found (e.g., agricultural and urbanized areas). In this paper, we aimed to evaluate the current and future potential European range of the two target species, taking into account their co-occurrence in different greenhouse gas emission scenarios. We took advantage of both ENM techniques, which are widely used for the study of biological invasions [18,52,53], coupled with GIS analyses, used as well for biodiversity and conservation issues [54,55]. Further, we refined the outcomes of the modelling process with the information about the areas where *A. artemisiifolia* is currently recorded (i.e., specific croplands and urbanized areas), selecting these territories through satellite remote sensing (SRS) data and geographic information system (GIS) spatial analyses at the European scale, considering a plausible future scenario, where a straight connection is established between the increase of allergies and the destiny of populations living in cities.

## 2. Materials and Methods

### 2.1. Study Species

In this study, we considered the following two species:

*Ambrosia artemisiifolia* L. (Asteraceae), native to North America, is an invasive plant species that has naturalized in most parts of the world, including Africa and Oceania. The common ragweed is a very competitive weed and can produce yield losses in many cultivations, especially in soybeans [56]. Its wind-blown pollen is highly allergenic to humans [47].

*Ophraella communa* LeSage, an oligophagous leaf beetle (Chrysomelidae, Galerucinae) originating from the Nearctic region, whose adults and larvae feed on the leaves of some plant species of the Asteraceae, including the common ragweed, *Ambrosia artemisiifolia* [57]. Adults appear from May to October and deposit eggs on the host leaves. Larvae eat leaves and complete their development in about 12 days. Mature larvae spin cocoons to pupate on the host. It takes about one summer month for one generation. The primary range includes the south-eastern area of North America, while the secondary range comprises some southern Asian (Japan and China) and European (Italy and Switzerland) countries [34,38].

### 2.2. Dataset and Study Area

For both the primary and secondary range, records for *Ambrosia artemisiifolia* and *Ophraella communa* were gathered by integrating GBIF occurrences and presence localities from published resources, for a total of 23,262 occurrence localities for *A. artemisiifolia* and 359 for *O. communa*; possible duplicate records were discarded through the ‘validate topology’ tool in ArcMap 10.0 [58]. To avoid any correlation among the remaining presence localities, a partial removal of occurrences was performed through the ‘spThin’ package [59] in R environment [60], setting the thinning parameter at 30 km and 10 replicates. Moran’s test was performed (‘spatial autocorrelation’ tool in ArcMap) for both the target species’ datasets to further test possible self-correlation among presence localities. The datasets used for the analyses are reported in the Supplementary Material Table S1. The study area was focused in Europe, which is one of the invaded ranges of the two species worldwide; the presence locations in Northern and Central America were used for the models’ calibration (see below).

### 2.3. Ecological Niche Modelling

To model current and future habitat suitability for both target species, the set of 19 bioclimatic variables at 30” resolution was downloaded from the online repository Worldclim.org [61]. Bioclimatic layers were cut to the geographic extent of both the native (North and Central America) and secondary range in Europe and processed through the ‘band collection statistics’ tool in ArcMap, for testing the correlation among predictors (Supplementary Material Table S2), considering a Pearson’s |r| < 0.85 [62,63].

To calculate models in future climatic scenarios, we chose three representative concentration pathways (RCPs), 4.5, 6.0, and 8.5, and three different global climate models for each RCP, namely the CCSM4 [64], the IPSL [65], and the MIROC-CHEM [66].

Ecological niche models (ENMs) were built using the Maxent algorithm [20] implemented in the “SDMtoolbox” 2.4 version [67] in ArcMap 10.0. This toolbox takes advantage of the powerful Maxent machine-learning algorithm, a modelling approach, integrating corrective files, and lowering possible biases, which may occur during model calibration and/or projection, during the data preparation and the process itself [67,68]. The latitudinal bias effect was corrected, in our analysis, through the “Bias File for Coordinate Data” tool, and the generation of pseudo-absences was improved through the “Background Selection: Sample by Buffered Minimum Convex Polygon (MCP)”; the resulting files were combined with the “Clip Bias File for Coordinate Data (BFCD) by the Background Bias File” tool.

Models were calculated through the “Run MaxEnt: Spatial Jackknifing” tool, using the bias file obtained with the previous steps. Variables from both the primary and secondary range were used for model calibration, and projections were made for the secondary range for the three future climatic scenarios considered [1,22]. Spatial jackknifing, for improving the reliability of Maxent predictions [68], was set at 5 spatial groups, with no thresholds set during this stage. The future projections resulting from each different general circulation model (GCM) were combined through the multivariate environmental dissimilarity index (MEDI) algorithm [69]. This procedure takes advantage of the multivariate environmental surface similarity (MESS) (the measure of models’ extrapolation with respect to the calibrating conditions) and proportionally averages the predictions resulting from the different GCMs, down-weighting models with higher differences compared to environmental conditions used for model calibration (and vice-versa). Predicted levels of suitability were divided in three classes: Low (1), medium (2), and high (3), with thresholds 33% ÷ 66% ÷ 99%, respectively, as also performed in other ENMs-based papers (e.g., [70,71,72]). This procedure was chosen to discretize the ENMs’ continuous output to facilitate a comparison between the two target species among the different time scenarios considered, avoiding a high number of combinations.

### 2.4. Post-Modelling Analyses

MEDI-corrected models were further processed in the GIS environment to refine these predictions in more plausible scenarios of distribution of the two study species. Thus, considering the information on the preferred habitats colonized by *A. artemisiifolia*, we extrapolated the corresponding croplands (see below) in the study area using SRS data. For this purpose, we used an object-based method [73,74,75] through the Google Earth Engine (GEE) cloud platform, by using the multispectral Sentinel-2 (at a 10-m spatial resolution) satellite recorded from 2016 to 2018. The GEE portal provides access to global time-series satellite imagery, vector data and other ancillary data, cloud-based computing, and algorithms for processing large amounts of data [76].

We performed latitudinal corrections to take into account the phenology of the crops more sensitive to the invasion of *A. artemisiifolia* [23,24,25]; the image collections were mediated and merged in a time interval ranging from April to October [77]. A false color composite (cloud free) for all of Europe consisting of SWIR wavelengths (2202.4 nm for S2A and 2185.7 nm for S2B), NIR wavelengths (835.1 nm for S2A and 833 nm for S2B), and BLUE wavelengths (496.6 nm for S2A and 492.1 nm for S2B) was created to set a pixel-value threshold used for the identification of agricultural patches through the classification method mentioned above. These data were further validated in the GIS environment, by comparing them with the “agricultural” categories (coded as “2”) of the Corine Land Cover (CLC) 2018 (III level).

Data were further refined by intersecting the above-mentioned dataset with the global food security-support analysis data at a 1-km spatial resolution (GFSAD1 km) [78], which contains the spatial distribution of the five main types of global farmlands (wheat, rice, corn, barley, and soy). Based on bibliographical information [23,24,25], we selected the agricultural classes preferred for *A. artemisiifolia*’s spread (classes 2 and 4 of the GFSAD1 km). From now on, these areas will be named ‘target-croplands’.

Other areas that favor the spread of *A. artemisiifolia*, namely urbanized areas and the corresponding roads and infrastructures [79,80], were considered in refining the models. These spatial data were obtained through the CLC 2018 level III.

Target croplands and urbanized areas were used for both current and future analyses. Although we are aware that these data will change, it will be difficult (if not impossible) for agricultural areas to diminish, or urbanized areas to shrink, in the near future. Therefore, all future predictions are a plausible underestimation of the potential future trends of invasion and resulting analyses can be acknowledged as a starting point for future studies.

All satellite data processes were managed through the GEE platform and SNAP 6.0 software, whereas all spatial processes and geographic analyses derived from the elaboration of both ENMs and satellite/GIS data were managed in ArcMap 10.0 [58].

## 3. Results

The ‘thin’ function [59] reduced the two datasets from 23,262 to 1084 points for *Ambrosia artemisiifolia*, and from 359 to 81 points for *Ophraella communa*. Moran test resulted in no autocorrelation among presence localities for both the species considered, with values of 0.0004 (z-score = 1.62, *p* = 0.109) for *A. artemisiifolia* and 0.0066 (z-score = 0.849, *p* = 0.396) for *O. communa*.

Based on the correlation matrix (Supplementary Material Table S2), the following 11 bioclimatic variables were selected to perform the modelling process: BIO1 (annual mean temperature), BIO2 (mean diurnal range), BIO6 (minimum temperature of the coldest month), BIO7 (temperature annual range), BIO8 (mean temperature of the wettest quarter), BIO10 (mean temperature of the warmest quarter), BIO11 (mean temperature of the coldest quarter), BIO12 (annual precipitation), BIO13 (precipitation of the wettest month), BIO15 (precipitation seasonality), and BIO18 (precipitation of the warmest quarter).

**Figure 1:**
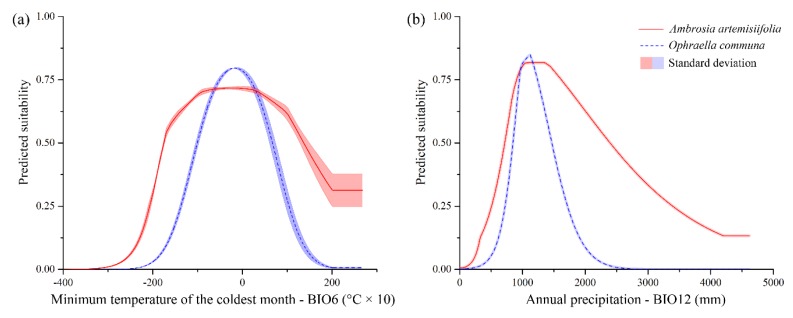
Shared variables’ response curves for *Ambrosia artemisiifolia* and *Ophraella communa*. Marginal response curves obtained for the two variables BIO6 (minimum temperature of the coldest month) (**a**) and BIO12 (annual precipitation) (**b**), which were among the three most contributive predictors for both *Ambrosia artemisiifolia* and *Ophraella communa*.

ENMs showed high values of the mean area under the curve (AUC) of the receiver operating characteristic curve (AUC = 0.871 for *A. artemisiifolia*; AUC = 0.966 for *O. communa*), and low values of standard deviation (SD) (SD = 0.003 for *A. artemisiifolia*; SD = 0.012 for *O. communa*) for the whole replicates. The three most contributing variables were: BIO6 (43.8%), BIO12 (25.3%), and BIO10 (20.3%) for *A. artemisiifolia*, and BIO12 (36.2%), BIO6 (21.3%), and BIO15 (16.7%) for *O. communa*. The marginal response curves obtained for the most contributing variables, BIO6 and BIO12, show different trends for the two species analyzed. For both variables, the ranges of *O. communa* are narrower with respect to the ones of *A. artemisiifolia*, which means they are more tolerant both to lower and higher values in the minimum temperature of the coldest month, and to higher values of the annual precipitation. However, the ranges of optimality in both cases are widely shared with *O. communa* (Figure 1). Thus, a wider adaptability was found for *A. artemisiifolia*, considering a high tolerance to low and high values in the minimum temperature of the coldest month, and to high values of annual precipitation.

**Figure 2:**
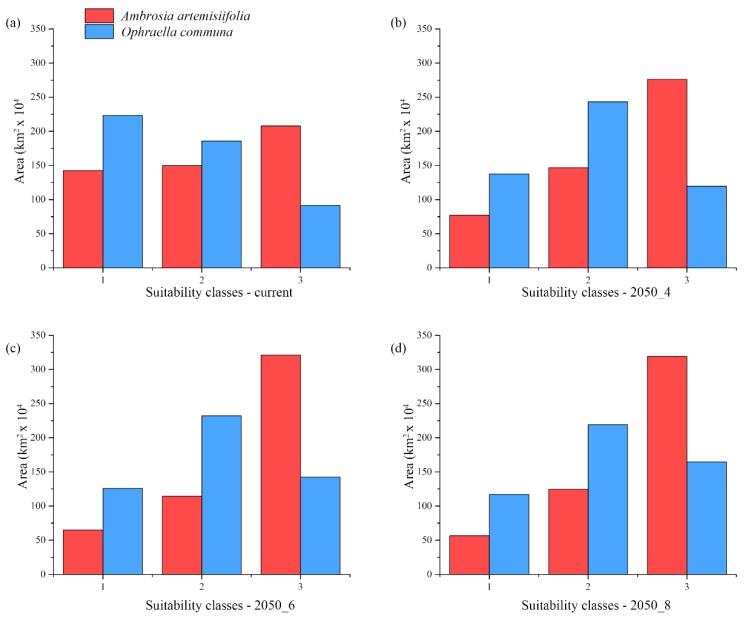
Suitable areas predicted for *Ambrosia artemisiifolia* and *Ophraella communa* for current and future climate scenarios. Predicted low (1), medium (2), and high (3) suitable areas for current (**a**), 2050—RCP 4.5 (**b**), 2050—RCP 6.0 (**c**), and 2050—RCP 8.5 (**d**) scenarios for *Ambrosia artemisiifolia* (red) and *Ophraella communa* (blue).

A wide area was predicted in medium and high classes for *A. artemisiifolia* for the current climatic conditions, while a less extensive area was predicted as suitable for *O. communa* in the same classes (Figure 2). For the future scenarios, the predicted suitability in medium and high classes increased for both species, with the higher classes of *A. artemisiifolia* and *O. communa* increasing with the increase of the RCPs considered (from 2050-RCP 4.5 to 2050-RCP 8.5). Nevertheless, *O. communa* showed: (a) A more limited extent of predicted suitable areas for the high class, which ranged from 43% to 51% with respect to the ones predicted for *A. artemisiifolia*; and (b) an opposite trend, instead, for the low (from 156% to 206%) and medium (from 123% to 202%) classes (Figure 2).

**Figure 3:**
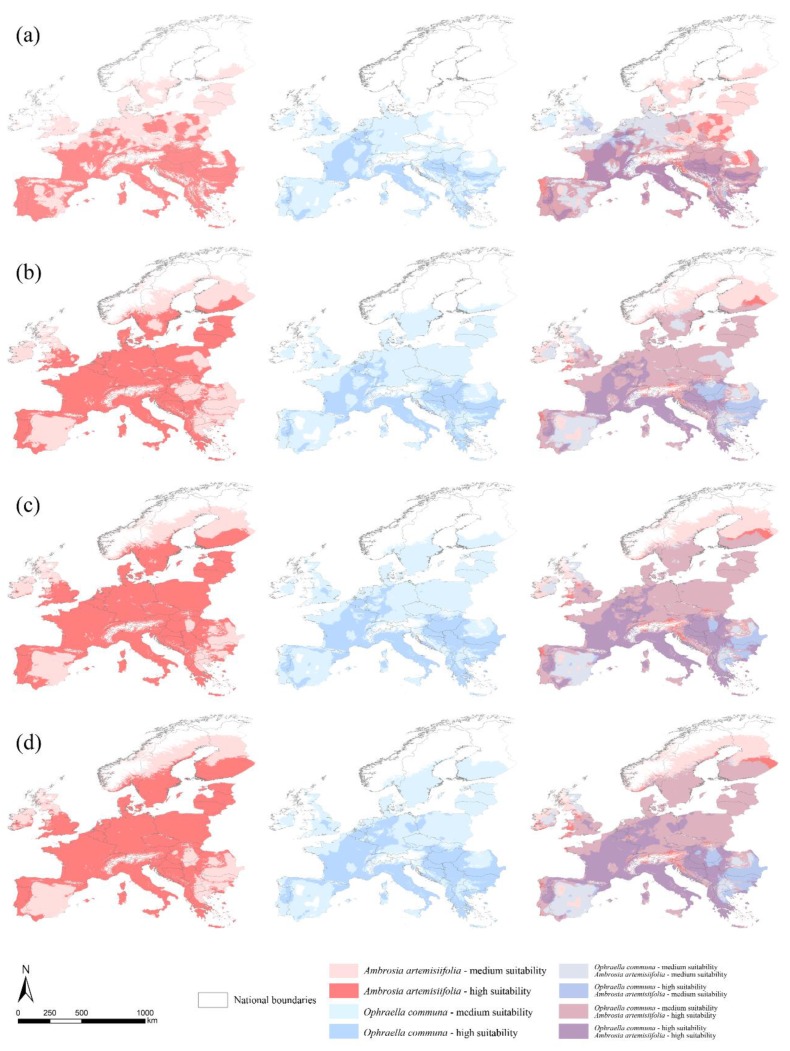
Maps of predicted suitability for *Ambrosia artemisiifolia* and *Ophraella communa* for current and future climate scenarios. Models of predicted suitability (low suitability areas are not displayed for graphical purposes; these correspond to white zones for both target species) obtained for *Ambrosia artemisiifolia* (red) and *Ophraella communa* (blue), and the overlap between them, for current (**a**), 2050—RCP 4.5 (**b**), 2050—RCP 6.0 (**c**), and 2050—RCP 8.5 (**d**) scenarios.

The current climate scenario shows continuous and high suitable areas for *A. artemisiifolia*, extended also in the north-western territories of Europe. On the contrary, *O. communa* shows a more fragmented scenario incompletely covering the suitable areas of *A. artemisiifolia*, mainly in the Balkans and north-eastern Europe (Figure 3a). In future scenarios, a more regular continuity among the separated areas of the current scenario is predicted for *A. artemisiifolia*, while for *O. communa*, we obtained changes in its range depending on the RCP considered (Figure 3b–d).

**Figure 4:**
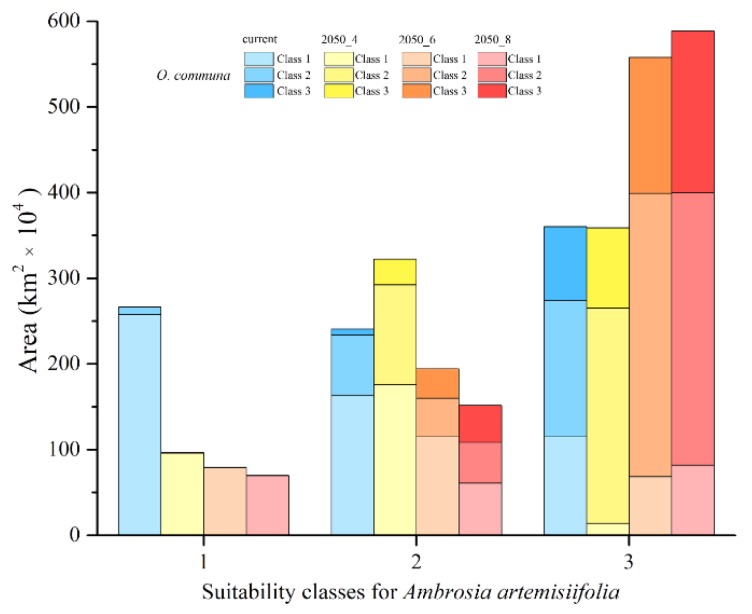
Shared predicted suitable areas for *Ambrosia artemisiifolia* and *Ophraella communa* for current and future climate scenarios. Areas (cumulative) shared by *Ambrosia artemisiifolia* and *Ophraella communa* in the three different suitability classes (class 1 = low; class 2 = medium; class 3 = high) predicted by the models, performed for each different time frame considered.

The overlap between the two species for medium and high classes of suitability increases, but several suitable areas for *A. artemisiifolia* are not suitable for *O. communa* in the medium and high classes. The greatest overlap occurs between the medium suitability class of *O. communa* and the high suitability class of *A. artemisiifolia*, highlighting how a potential biocontrol by *O. communa* will probably not be able to limit the spread of *A. artemisiifolia* (Figure 4).

To infer possible biological control exerted by *O. communa* over *A. artemisiifolia*, the area shared by the two species was calculated and intersected considering two land-use typologies, croplands (soybean, sunflower, maize, and sugar beet) obtained by satellite data, and urbanized areas by CLC 2018. For the current scenario, a total of 92.97% of the target croplands intersects the areas with medium (51.36%) and high (41.61%) classes of suitability for *A. artemisiifolia*; the value rises to 97.06% for the urbanized areas.

**Figure 5:**
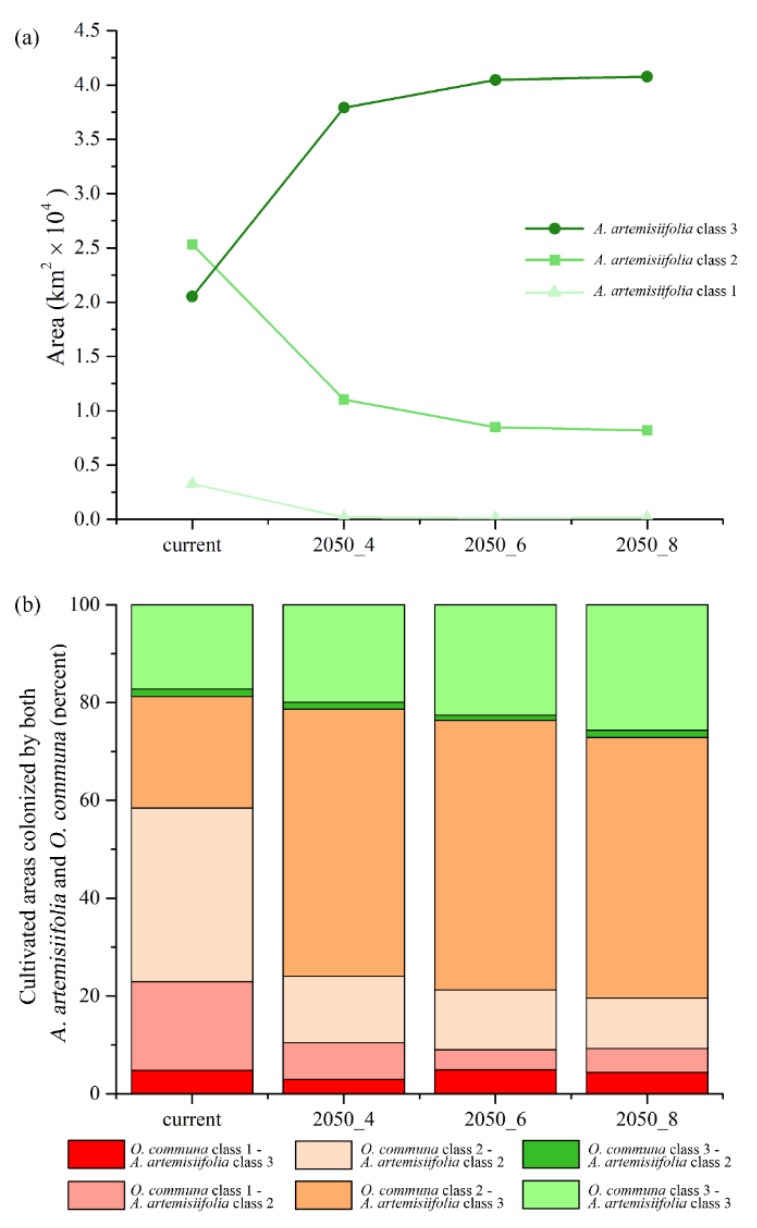
Trends for *Ambrosia artemisiifolia* and cumulative areas shared by target species in agricultural areas. Areas predicted as suitable (class 1 = low; class 2 = medium; class 3 = high) for *Ambrosia artemisiifolia* falling within target agricultural patches in the different time frames considered (**a**); areas (percent, cumulative) potentially shared by both *Ambrosia artemisiifolia* and *Ophraella communa* for the different suitability classes (class 1 = low; class 2 = medium; class 3 = high) and time frames considered, falling within target agricultural patches (**b**).

**Figure 6:**
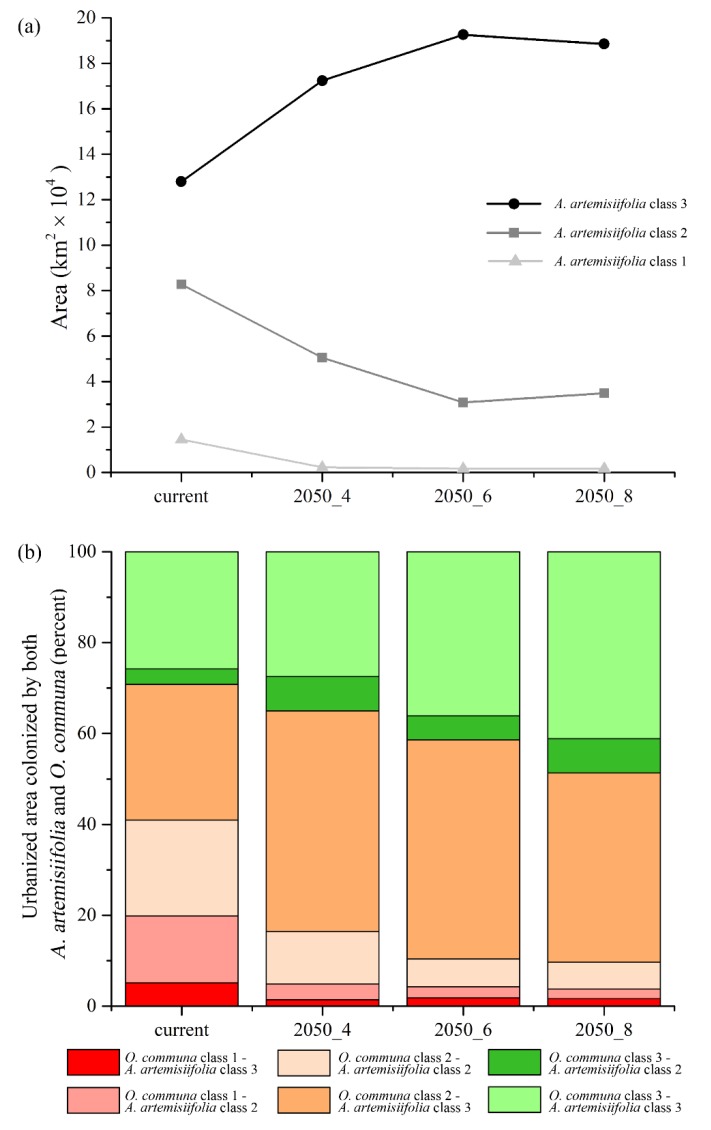
Trends for *Ambrosia artemisiifolia* and cumulative areas shared by target species in urbanized areas. Areas predicted as suitable (class 1 = low; class 2 = medium; class 3 = high) for *Ambrosia artemisiifolia* falling within urbanized patches in the different time frames considered (**a**); areas (percent, cumulative) potentially shared by both *Ambrosia artemisiifolia* and *Ophraella communa* for the different suitability classes (class 1 = low; class 2 = medium; class 3 = high) and time frames considered, falling within urbanized patches (**b**).

For future scenarios, an increase of the highly suitable areas is predicted from the 2050-RCP 4.5 to the 2050-RCP 8.5, ranging from +84% to +98% for target croplands, and from +34% to +50% for urbanized areas (Figure 5a and Figure 6a). On the contrary, a concurrent decrease for low and medium classes of suitability is observed (Figure 5a and Figure 6a).

In the current scenario, many cultivated territories host medium or high suitable conditions for both species (medium suitability for *O. communa*; high suitability for *A. artemisiifolia*, 22.7%; and medium suitability for both, 35.5%) (Figure 5b), while for future scenarios, a change is observed for the shared areas. An increase of high suitable conditions for *A. artemisiifolia* and medium suitable conditions for *O. communa* is evident in cropland areas for all the RCPs considered (54.6% in 2050-RCP 4.5, 55.2% in 2050-RCP 6.0, and 53.2% in 2050-RCP 8.5), as well as a slight increase of croplands hosting high climatic suitability for both the target species (19.9% in 2050-RCP 4.5, 22.6% in 2050-RCP 6.0, and 25.6% in 2050-RCP 8.5) (Figure 5b). Still, in the croplands, a contraction of areas with medium suitability for both species is also observed (12% on average), and a stability of shared areas with low suitability for *O. communa* and high suitability for *A. artemisiifolia* is also predicted from RCP 4.5 to RCP 8.5 (Figure 5b).

Moreover, comparable trends with respect to the selected croplands are also observed for the shared areas between *A. artemisiifolia* and *O. communa* overlapping urbanized areas, with increasing percentages of medium suitability for *O. communa*, high suitability for *A. artemisiifolia*, and high suitability for both (~45% and 27% ÷ 41%, respectively), and a decreasing trend for medium suitability areas (11% ÷ 5%) (Figure 6b).

**Figure 7:**
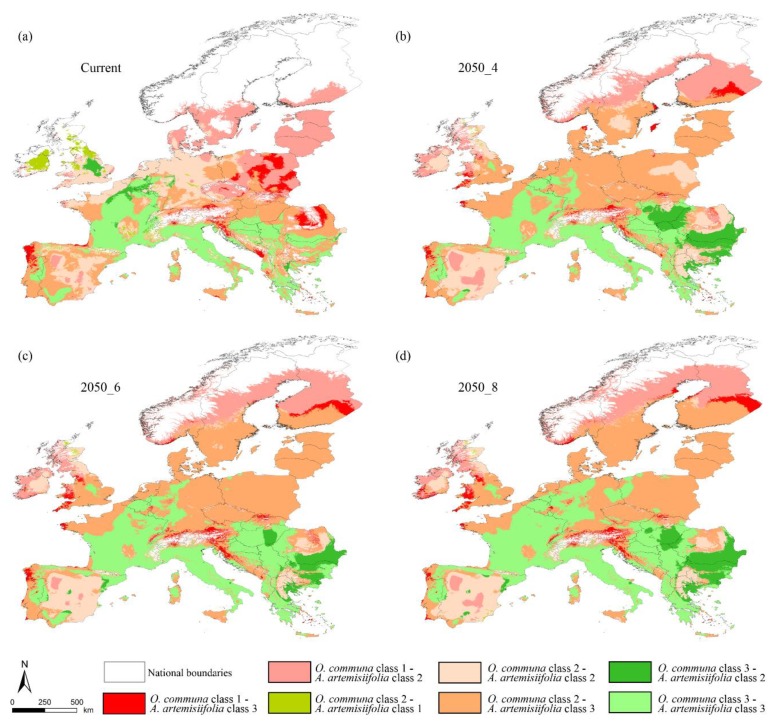
Territories of overlapping classes of suitability (class 1 = low; class 2 = medium; class 3 = high) for *Ambrosia artemisiifolia* and *Ophraella communa* in the different time frames considered ((**a**) current; (**b**) 2050 RCP 4.5; (**c**) 2050 RCP 6.0; (**d**) 2050 RCP 8.5).

Finally, the co-occurrence analyses performed over European countries depict a current scenario (Figure 7a) where northern France, central Belgium, central Great Britain, and northern Greece show the ‘best’ co-occurrence combination (*O. communa* class 3—*A. artemisiifolia* class 2) for biocontrol of the *A. artemisiifolia* invasion, while the ‘worst’ scenario (*O. communa* class 1—*A. artemisiifolia* class 3) is observed in the north of Portugal and of Spain, southern Montenegro, northern Albania, central Romania, and vast areas of central and southern Poland. In the future predictions, an increase in the overlap between the target species is observed in different countries with respect to the predictions of the current scenario. In fact, the ‘best’ scenario is inferred throughout Romania, Bulgaria, and vast areas of Serbia and Hungary. On the contrary, the ‘worst’ scenario is predicted for southern Finland, northern Portugal, and western U.K. (Figure 7b–d). About medium suitability classes for both target species, a general shift to the ‘*O. communa* class 2—*A. artemisiifolia* class 3′ is observed with respect to the current scenario, where a ‘mix’ between ‘class 2—2′ and ‘class 2—3′ are predicted for central and northern Europe and the Iberian peninsula (Figure 7).

## 4. Discussion

The ecological difference found between the climatic preferences of two target species reveals lower adaptation capabilities for *O. communa* with respect to its host plant *A. artemisiifolia*. The wider tolerance for low temperatures and precipitation variability offers this plant a considerable advantage in potentially occupying territories in the European area of the secondary range. The highly suitable areas for *O. communa* seem to be not enough to stem the corresponding areas of *A. artemisiifolia*, as also reported by Sun, Brönnimann, Roderick, Poltavsky, Lommen, and Müller-Schärer [50] for Asian areas. Indeed, suitable climatic conditions are spread throughout Europe in all time frames considered, even though with an evident prevalence of higher suitable classes of *A. artemisiifolia* intersecting (i.e., not potentially controlled by) the ones of *O. communa*. The control that this leaf beetle exerts over the invasive plant is of primary importance because of the problems that *A. artemisiifolia* causes to agriculture and human health [81,82,83].

A general increase in the higher class of climatic suitability for this species is predicted to occur in both target croplands and urbanized areas in future scenarios, which is predicted to happen to the exclusion of the low and medium classes (1 and 2, respectively) (Figure 5a and Figure 6a). The increase of these areas goes along, for both of these land cover/land use categories, with the increase of greenhouse gas emissions (i.e., the growing radiative forcing of the RCPs), indirectly confirming that the increase of CO_2_ favors the spread of this invasive plant [43].

The high suitable area of *A. artemisiifolia* intersecting the medium suitable areas of *O. communa* is a remarkable trend resulting from our analyses. These possible future assets would then result in a more efficient spread of *A. artemisiifolia* coupled with an increasing difficulty in the biocontrol by *O. communa*, confirming the results obtained in other invaded areas [36]. This trend is mainly observable in Germany, Netherland, Denmark, southern Sweden and Finland, Czech Republic, Slovakia, Poland, Lithuania, Latvia, Estonia, and part of the U.K., France, and Portugal. These countries will face a wide potential spread of *A. artemisiifolia* on which *O. communa* will not be able to exert control. The future scenario predicted for Romania, Bulgaria, France, Italy, Croatia, Serbia, Albania, and Greece reveals the possibility of strong biocontrol. These countries could start massive rearing of *O. communa* for this purpose, considering the high climatic suitability of their territories, thus encouraging this biocontrol practice as a management action going along with the other ones (e.g., mowing, herbicide treatments) suggested in the literature. Indeed, this practice should consider the genetic variation in performance traits [84,85], which could cause different population feedbacks in response to environmental conditions of the current and future colonized areas.

Considering our analyses, the use of *O. communa* as a control agent is desirable, even though some other *A. artemisiifolia* biological control agents were identified [29,39,50]; further research is needed in terms of coupled ENMs and SRS data to obtain more accurate estimates of the current and future assets, especially for in-detail territorial strategies. Finally, the use of land cover future projections would further improve the methodological framework we proposed in this paper, to more accurately address decision makers’ policies.

## 5. Conclusions

The integrated use of ecological niche modelling and satellite remote-sensing techniques in the GIS environment, as performed in our study, was shown to offer some advantages in the study of the potential distribution of IAS. This approach permits assessment of the potential biocontrol exerted by a target species before practical managing actions’ start, focusing economical efforts on strategies for specific areas. The categorization of climatic preferences helps to highlight land cover patches of particular interest for invasive species, reducing the uncertainties of a sole SDM approach. The use of SRS permits an accurate post-modelling analysis to be performed at relatively low costs (given the increased availability of open source data). Furthermore, this approach allows detection and quantification of the possible land cover patches affected by the invasion of a target IAS so as to better address management actions, especially in consideration of the social and economic issues that IAS may bring.

## Supplementary Materials

The following are available online at https://www.mdpi.com/1660-4601/16/18/3416/s1, Table S1: Table reporting localities used for the analyses, with the corresponding coordinates in decimal degrees, rounded to three decimal places (datum: WGS84). Table S2: Correlation matrix calculated for the 19 candidate predictors.
