# Supplementary material for: Investigating the Current and Future Co-Occurrence of Ambrosia artemisiifolia and Ophraella communa in Europe through Ecological Modelling and Remote Sensing Data Analysis
Source: Int J Environ Res Public Health. 2019 Sep 14;16(18):3416. doi: 10.3390/ijerph16183416 (PMC6766007; doi:10.3390/ijerph16183416)
Supplement: Supplementary file 1 [file ijerph-16-03416-s001.zip › Table_S2 - Iannella et al 2019_Ophraella.docx]

**Investigating the current and future co-occurrence of *Ambrosia artemisiifolia* and *Ophraella communa* in Europe through ecological modelling and remote sensing data analysis**

Mattia Iannella^1^, Walter De Simone^1*^, Paola D’Alessandro^1^, Giulia Console^1^, Maurizio Biondi^1^

**Supplementary File 2 – Correlation matrix calculated for the 19 candidate predictors**

|  | **BIO1** | **BIO2** | **BIO3** | **BIO4** | **BIO5** | **BIO6** | **BIO7** | **BIO8** | **BIO9** | **BIO10** | **BIO11** | **BIO12** | **BIO13** | **BIO14** | **BIO15** | **BIO16** | **BIO17** | **BIO18** | **BIO19** |
| --- | --- | --- | --- | --- | --- | --- | --- | --- | --- | --- | --- | --- | --- | --- | --- | --- | --- | --- | --- |
| **BIO1** | 1.00 | -0.21 | -0.45 | 0.23 | 0.16 | -0.28 | 0.82 | 0.08 | 0.50 | 0.12 | -0.65 | 0.24 | 0.73 | 0.24 | 0.32 | 0.83 | 0.75 | 0.52 | 0.49 |
| **BIO2** | -0.21 | 1.00 | 0.33 | -0.12 | -0.08 | 0.31 | 0.04 | 0.08 | 0.45 | -0.08 | 0.36 | -0.09 | -0.09 | -0.12 | 0.15 | 0.11 | 0.35 | 0.55 | -0.38 |
| **BIO3** | -0.45 | 0.33 | 1.00 | -0.40 | -0.44 | -0.12 | -0.69 | 0.23 | 0.35 | -0.43 | 0.90 | -0.39 | -0.27 | -0.45 | 0.48 | -0.54 | -0.25 | 0.18 | -0.55 |
| **BIO4** | 0.23 | -0.12 | -0.40 | 1.00 | 0.60 | 0.56 | 0.34 | 0.06 | -0.07 | 0.56 | -0.39 | 0.99 | 0.23 | 0.92 | -0.13 | 0.31 | 0.22 | 0.04 | 0.85 |
| **BIO5** | 0.16 | -0.08 | -0.44 | 0.60 | 1.00 | 0.71 | 0.29 | -0.65 | -0.17 | 0.99 | -0.35 | 0.59 | 0.08 | 0.85 | -0.25 | 0.24 | 0.16 | -0.02 | 0.62 |
| **BIO6** | -0.28 | 0.31 | -0.12 | 0.56 | 0.71 | 1.00 | -0.02 | -0.33 | -0.18 | 0.72 | 0.00 | 0.55 | -0.15 | 0.68 | -0.23 | -0.03 | 0.00 | -0.03 | 0.22 |
| **BIO7** | 0.82 | 0.04 | -0.69 | 0.34 | 0.29 | -0.02 | 1.00 | 0.01 | 0.44 | 0.25 | -0.80 | 0.35 | 0.68 | 0.35 | 0.11 | 0.97 | 0.86 | 0.56 | 0.47 |
| **BIO8** | 0.08 | 0.08 | 0.23 | 0.06 | -0.65 | -0.33 | 0.01 | 1.00 | 0.30 | -0.67 | 0.05 | 0.08 | 0.27 | -0.28 | 0.40 | 0.10 | 0.15 | 0.21 | -0.12 |
| **BIO9** | 0.50 | 0.45 | 0.35 | -0.07 | -0.17 | -0.18 | 0.44 | 0.30 | 1.00 | -0.20 | 0.08 | -0.04 | 0.54 | -0.11 | 0.74 | 0.59 | 0.81 | 0.95 | -0.07 |
| **BIO10** | 0.12 | -0.08 | -0.43 | 0.56 | 0.99 | 0.72 | 0.25 | -0.67 | -0.20 | 1.00 | -0.33 | 0.55 | 0.04 | 0.81 | -0.28 | 0.20 | 0.11 | -0.06 | 0.58 |
| **BIO11** | -0.65 | 0.36 | 0.90 | -0.39 | -0.35 | 0.00 | -0.80 | 0.05 | 0.08 | -0.33 | 1.00 | -0.39 | -0.65 | -0.41 | 0.06 | -0.74 | -0.44 | 0.00 | -0.54 |
| **BIO12** | 0.24 | -0.09 | -0.39 | 0.99 | 0.59 | 0.55 | 0.35 | 0.08 | -0.04 | 0.55 | -0.39 | 1.00 | 0.24 | 0.91 | -0.11 | 0.33 | 0.25 | 0.06 | 0.84 |
| **BIO13** | 0.73 | -0.09 | -0.27 | 0.23 | 0.08 | -0.15 | 0.68 | 0.27 | 0.54 | 0.04 | -0.65 | 0.24 | 1.00 | 0.18 | 0.70 | 0.79 | 0.70 | 0.46 | 0.32 |
| **BIO14** | 0.24 | -0.12 | -0.45 | 0.92 | 0.85 | 0.68 | 0.35 | -0.28 | -0.11 | 0.81 | -0.41 | 0.91 | 0.18 | 1.00 | -0.20 | 0.32 | 0.22 | 0.03 | 0.85 |
| **BIO15** | 0.32 | 0.15 | 0.48 | -0.13 | -0.25 | -0.23 | 0.11 | 0.40 | 0.74 | -0.28 | 0.06 | -0.11 | 0.70 | -0.20 | 1.00 | 0.32 | 0.44 | 0.54 | -0.15 |
| **BIO16** | 0.83 | 0.11 | -0.54 | 0.31 | 0.24 | -0.03 | 0.97 | 0.10 | 0.59 | 0.20 | -0.74 | 0.33 | 0.79 | 0.32 | 0.32 | 1.00 | 0.93 | 0.68 | 0.41 |
| **BIO17** | 0.75 | 0.35 | -0.25 | 0.22 | 0.16 | 0.00 | 0.86 | 0.15 | 0.81 | 0.11 | -0.44 | 0.25 | 0.70 | 0.22 | 0.44 | 0.93 | 1.00 | 0.89 | 0.26 |
| **BIO18** | 0.52 | 0.55 | 0.18 | 0.04 | -0.02 | -0.03 | 0.56 | 0.21 | 0.95 | -0.06 | 0.00 | 0.06 | 0.46 | 0.03 | 0.54 | 0.68 | 0.89 | 1.00 | 0.01 |
| **BIO19** | 0.49 | -0.38 | -0.55 | 0.85 | 0.62 | 0.22 | 0.47 | -0.12 | -0.07 | 0.58 | -0.54 | 0.84 | 0.32 | 0.85 | -0.15 | 0.41 | 0.26 | 0.01 | 1.00 |
